# Prize-Hospital Report, No. V

**Published:** 1828-01-01

**Authors:** 


### PRIZE HOSPITAL REPORT, No. V.

MR. G. WICKHAM, GUY'S HOSPITAL.

### I.

### A CASE OF "DELIRIUM TREMENS" TERMINATING IN "IDIOTISM."

THOMAS ROLFE, æt. 29, a shoe-maker, was admitted, under Dr. Elliotson, 21st July, with symptoms of muscular debility, amounting almost to paralysis. There is trembling of the extremities, so that he is incapable of holding any thing steadily, or even of standing quietly—he is thin, and appears to have lost flesh. On being questioned as to the probable cause of his complaint, he betrays some incoherence, as if induced by mental distress, constantly referring to circumstances of business, without shewing the slightest connexion. He distinctly speaks of pain in the head, when asked concerning it, but every part of the body is equally the seat of pain—his genitals, however, seemed to draw his attention considerably, although he could describe neither the pain itself, nor the cause for it. A relative informs us that this state has been eight or nine weeks from its commencement; he has been occasionally attacked with spasms, as she termed it, which once terminated in a temporary loss of sense and power; he has been harrassed in business very considerably, and has laid his head upon a pillow at the end of the day, as if more perplexed than fatigued; he has been married several years, but has no children.

Baln. tepid. at 95° b. d. for 15 minutes. Emp. canth. fronti et occipiti, under the idea that pain existed in the head to some extent, which we learnt from the wife afterwards, he had never complained of. Acid. Prussici, ℳ iij. Infus. sennæ, ℥j. ter die; strong beef tea, ℔iij. quotidie.

23*d*. Pulse varies from 72 to 84—stools copious and dark. There is still universal pain, if asked concerning it. He flinches on pressure at the pit of the stomach alone. Acid. Pr. ℳ jv. Inf. sennæ, ℥ss. t. d.

26*th*. Debility very great—tremor much increased—incoherence and jactitation both very prominent—restlessness during the night. These induced the opinion of its being *delirium tremens*. Pulv. opii, gr. ij. ter die.

28*th*. Much quieter, and has slept. Pulv. opii, gr. iij. ter die.

30*th*. Better in every respect—trembling almost gone—slept well—incoherence concealed from a reluctance to talk—pulse 70 and regular—not increased in volume. Pulv. opii, gr. jv. Inf. sennæ, p. r. n. Has been purged, but is better.

*July 3d*. Perfectly quiet as to trembling, but very restless and incoherent—bowels open—pulse full and soft: has taken senna daily. Opii, gr. v. t. d. Mutton chop alt. die.

7*th*. The opium has been increased to eight grains without affecting the system, but his intellects are gradually losing power.

19*th*. He has remained in the hospital till to-day, although his present state is confirmed *idiotism*. He is now removed to Bethlem Hospital.

Making every allowance for abstract terms, I should be inclined to designate the commencement of this case, a *first stage* merely of idiotism. 'Tis true, there was "equal feebleness of body and mind," but the term *delirium tremens* is referable to that state only, when following "too free a use of spirituous liquors," a propensity to which was denied, in this case, by all his friends. But, according to Mason Good, "violent agitation of the passions" is not an unfrequent cause of "*accidental* idiotism," and "deep and protracted grief" seems here to have induced—for, being a man of very ordinary capacity and well disposed, he felt the misfortunes of life degrading to him, and hence the cause of anxiety, exertion, and, at last, distraction. His present state is a vague, unsteady, wandering eye, seldom fixed for any length of time upon any object; a stupid expression of countenance, in which a melancholic cast is mixed up; a spitting and increased secretion of saliva; a perpetual rolling of the head and tossing of the arms, legs, and the whole body; no memory, no language, no reason. It is said, that an "idiot has all the animal instincts and some of the passions:" but, of the latter, *joy*, *fear* and *anger*, seem those only with which he is affected, and these to a very limited extent. His joy is evinced by a voracious gratification of hunger and thirst—his fear, by quiet, after a threat merely, or strong language—and his anger, by a temporary fit of violence, when he chatters loudly, resists every thing, and attempts to bite any one in his way. Dr. Elliotson attributes the diarrhœa to the large doses of opium, which is by no means a singular circumstance.

---

### II.

### CASE OF EPILEPSY FROM ONANISM.

WM. BENNION, æt. 20, had been attending as an out-patient under Dr. Roots, and was admitted on the 31st of May, under Dr. Elliotson. Both agreed that he was labouring under epilepsy from onanism, which the lad unhesitatingly confessed to have practised, two or three times a day, till within these few months, when, for the first time, he had connexion with woman. The fits have been constant attendants on him for 18 months, varying in number from 3 to 11 a day. He is pale and emaciated—sensible of a weight in his head—his memory is so inconstant that he can scarce recollect words to express common observations: has been affected with a failure of sight and a ringing in his ears—he is constantly writing, and has a rapid succession of unconnected, mock-poetical ideas, which float before him, more like the fantasies of a wandering and delirious brain, than the steady effusion of a healthy imagination; every other mental function seems influenced by the same debility. Dr. Elliotson considers, that the organ of *ideality* is largely developed, but its exemplification is but strangely shewn in common with the other features of an idiot's character—than which he, at present, can scarcely be deemed much better.

The sulphate of zinc is the only remedy which was adopted at first, under which he evidently improved, having commenced with one grain, which was carefully increased to xvij. This afterwards was withdrawn, and the cupr. ammon. was substituted. The fits now rarely amount to more than three in the day, and are so slight, that he is conscious of what passes during their occurrence, and will run, at the moment of their cessation, to another part of the ward in quest of a glass to see the alterations of his countenance.

---

### III.

### FEVERS.

Four cases of Inflammatory Fever were admitted under Dr. Elliotson, 12th July, 1827. None of them differing in the essential symptoms, the same active measures were adopted, and, but for a relapse in one, were equally successful. The decisive practice of Dr. E. is well known, but the success of that decision cannot be better shewn than in the following cases.

WM. BURNETT, æt. 29, said that three weeks previous to admission, he was seized, while at work, with a sudden pain in the head, which, accompanied with shivering and vomiting, soon went on to delirium. He was bled and leeched on the temples, and is evidently better; he complains, at present, of tinnitus aurium and great depression of mental and bodily strength—few nights are unattended with wanderings and delirium. His pulse is quick and sharp, his tongue brownish, skin hot, eyes unsteady, and his countenance anxious.

Hyd. submur. gr. v. t. d. Emp. canth. fronti et occipiti.

*17th.* Purged: neither head-ache nor delirium left—mouth sore, and pulse expanded. Omit the calomel—cold ablution.

*21st.* Garg. sodæ chlorat.; the mouth being still very sore.

*24th.* Delirium returned—with aggravation of all the former symptoms. ℞. Vini antim. tart. ℨss. c. M. potassæ citratis, 6tis horis.

*25th.* Continued delirious at intervals —bowels have been confined, but are now relieved from ol. ricini, ℥j.

*27th.* Still unable to answer with any consistency—flinches on being pressed at the scrobiculis cordis—skin hot—tongue brown, and pulse firm. C. c. temp. ad ℥xij. Emp. canth. scrob. cordis. Hydr. c. cretâ, gr. v. 6tis horis.

*2d Aug.* State of mind wavers much, delirium being only intermitted by such a state of susceptibility that neither light nor sound can be borne—great debility. Hirud. iij. temp. retriq.

*4th.* Pulse weaker, though still hard—coma and delirium increased. Cal. gr. j. c. opio, gr. ¼, o. n. Ext. coloc. c. gr. x. alt. q. aurorâ—Canth. vesicat.

From this report, there was no deviation from a gradual sinking of the vital powers—his water has been drawn off three times a day—his fæces passed involuntarily—he could not express even what he wanted, and his pulse fell until the 7th, when he expired.

John Lowrey, æt. 21, had been ill 10 days, and complained of pain and giddiness of the head, with dimness of sight and distraction from the least sound—he has delirium occasionally—the pupils were dilated—the tongue foul, and white at the centre, and red at the edges—firm pulse—hot skin—no pain at the abdomen —has vomited, and been bled.

Hirud. xij. temporibus—Emp. canth. occipiti—Lot. ammon. acet. capiti. Hyd. submur. gr. v. 6 q. q. horâ.

*14th.* Skin very hot at times: cold ablution when needed—omit the calomel, mouth being sore.

*17th.* Tongue clean—no pain or delirium—three solid stools.

*27th.* Convalescent. Pulv. rhei, c. hyd. gr. xv. cras mane.

James Sheen, æt. 27, was admitted in a high state of delirium. Fever was well marked. His history was obtained from the friends, who stated that he had been ill for a fortnight and two days in the present raging state. There is pain in the epigastrium—pulse quick and hard. Hirud. viij. temp. et viij. epigastrio. Hyd. submur. gr. v. t. d.

*14th.* Omit the calomel. Hydr. c. cretâ, gr. x. t. d. Cold ablution when hot. Hirud. ut antè.

*17th.* Great relaxation of the bowels. Omit the hyd. c. cretâ.

*27th.* Symptoms perfectly relieved—some debility. Inf. gent. c. t. d.

*31st.* A relapse—some heat of skin and pain in abdomen, with diarrhœa. Mist. pot. citratis, c. Vin. antim. tart. ♏ xv. 6 q. q. horâ; Hydr. c. cretâ, gr. viij. b. d.; Opii, gr. ss. 4 q. q. horâ.

*7th August.* Symptoms again subdued. Sago c. syrup.

Johannah Sheen, æt. 15, complained of pain in her head, with dullness and vertigo. She groans constantly, and flinches when the abdomen is pressed—tongue is dry and brown—pulse 160, small and weak.

Hydr. submur. gr. v. t. d.; Fomentations to hands and feet; Hirud. xij. abdomini.

*14th.* Abrad. capil. et lot. ammon. acet. applied. Omit the calomel.

*24th.* Garg. rosæ.

*27th.* Convalescent—mouth still a little sore. Lot. plumb. subacet. dil. pro garg.

---

### IV.

### SLOUGHING PENES.

Wm. Skelton, æt. 21, was admitted 31st May, under Mr. Green, with a sloughing penis. He tells us that, three weeks ago, he first observed a discharge from under the prepuce, unattended with ardor urinæ, or any inconvenience beyond a little hardness and tenderness about the corona glandis. Cold water was his only remedy, and, about a fortnight afterwards, the prepuce began to swell and become very painful. No advice being obtained, the redness was soon converted into a black colour, which has but little altered up to the present hour. There is now very great tumefaction of the whole penis, with redness, heat, and pain; the prepuce projects over the glans, and by its swelling retains the water on his attempt to pass it. On the right side and upper part, there is a black slough, larger than a crown-piece, somewhat circumscribed, and indeed separated in one direction, but in another there appears a disposition to spread; the dark colour of the slough, and the redness of the surrounding inflammation, being intermixed.

Pulse quick, and somewhat hard—skin moderately cool—little thirst, and bowels regular. From his account, excitement has run much higher, especially before the slough was circumscribed. He was at the time compelled to keep his bed. M. s. c. statim. F. D.

*June 2d.* Has rested well each night, and is in but little pain—pulse lowered, and scarce any disturbance is observable—the slough is separating, and that appearance of spreading is now changed into a distinct limit.

*6th.* The slough came away on the 4th, and the wound is granulating. There is but a small lump of the prepuce left, which, from being thickened, must be very inconvenient—it was consequently removed. He was presented well.

John Vokes, æt. 32, was admitted, 31st May, under Mr. Green, with an immense destruction of his penis. The appearance is as if a section of the penis had been made, by which the glands and half the body had been lost. The surface is covered with a foul offensive discharge, interspersed with small spots of coagulated blood—the edges are irregular and scolloped. He states, that eight months ago he was affected with gonorrhœa, which was allowed to run its course without medicine. About two months afterwards he found his prepuce swelling, which, from his continuing his work, increased, gave pain, and excited some fear of its danger. Medical aid was obtained, but, from neglect afterwards, the part went on gradually to slough, each morning shewing a fresh loss of substance.

The pulse is weak and quick—he has the appearance of having lost much flesh, and is at present in a state of great debility, and unfit for work—his bowels are confined—skin moist, and violent perspiration is excited upon the slightest exertion.

Pulv. rhei, c. hyd. gr. xij. statim. Pulv. ipecac. c. gr. v. 6ta q. q. h. Cat. lini. F. D.

*6th June.* Pulse weak—appetite good—rests well, and bowels regular. Porter ℔j. quot.

*13th.* Wound clearing from sloughs—still weak. Ext. sarsæ, ʒss. Ex. decoct. ejusd. ℥iv. ter die.; Acid. nit. lot.

*16th.* Very much improved—he wished to go out, promising to return, if that improvement did not continue.

The former case, from its immense destruction, shews the necessity of speedy and active measures, in the onset of certain forms of slough; for, notwithstanding the great loss, (which thus might have been prevented,) and constitutional excitement, which gave rise to it, little or no *debility* seemed to exist; while in the latter case, *debility* had been the source of extension to the disease, wherein a supporting plan was serviceable. The contrast between the constitutional powers during the existence of destructive inflammation, is all I wish to preserve; for those conditions, in the present cases, are inferred from the local appearances alone, which, from analogy with other cases, are perfectly adequate to be our guide in treatment, and, indeed, almost without regard to any other symptoms. In one, there was a slow disorganization and absorption of the part, without apparent power either to resist that destruction, or restore its loss; but, in the other, the only source of extension seemed an *excess* of power, as indicated by the active inflammation surrounding the part, and the slough being in one mass, and yet to be thrown off. These form interesting varieties in our foul wards.

The following are likewise cases from our foul wards, and such as Mr. Green is constantly pointing out to us as *incurable*, except by mercury. It will be seen that they are secondary symptoms, *i. e.* following chancre; and it will be seen, also, that those symptoms have been temporarily cured once, at least. It is true, there is no evidence that the present plan will be more successful, but I have selected these as analogous cases to those which Mr. G. assures us *have returned repeatedly until they have undergone a course of mercury, but not afterwards.* I cannot think any one will project the mistaken idea, that the uncertainty of their return renders proof impossible; for, while "*I do not distinctly speak of proof*," there ought to be some instance, in the course of practice, in which mercury, given with satisfaction to the *surgeon*, had not permanently cured; especially when we consider that patients return with a repetition of their symptoms, *until* mercury has been properly exhibited—of which the following cases are not uncommon examples.

Mary Wilson, æt. —, was admitted under Mr. Green, 12th July, with a foul ulcer, involving the tonsil, arch of the fauces, and uvula.

Her history is, that nine months ago she had a sore upon the labium of a suspicious origin, which healed without the use of mercury, but that, shortly afterwards, a sore throat appeared, for which she gained admission into this hospital, took mercury, and went out cured; but it has again made its appearance. Now, upon speaking to Mr. Green, we learn, that the sore throat became speedily well under mercury, and that she left the hospital without that remedy being carried to its proper length. On this account the same plan has been adopted again; but the sore throat has again got well, and she, in like manner, has again left the hospital. Now, although such ingratitude and irregular conduct should prohibit any farther benefit from being held out to her, yet Mr. G. to establish his opinion, which may be useful to many others, has desired, upon her return, which he deems almost certain, that she may be re-admitted.

James Godfrey, æt. 26, was admitted 31st May, 1827, under Mr. Green, with several crusted eruptions upon his body, which are dark, elevated, and, for the most part, circular. On the head there is a deep ulcer, exposing the pericranium: its edges are rounded and callous—its surface flat and pale. This, he tells us, first shewed itself in a crust, which scaled off, and left a sore—it became gradually deeper, until it arrived at the present state.

Nine months ago, he states, he had a chancre, for which he took mercury, but to what extent is by no means clear—certainly not sufficiently long. The sore healed, and shortly afterwards he was attacked with sore throat and blotches, for which he gained admission at this hospital; and, being in a bad state of health, was treated with sarsaparilla and Plummer's pill. He was presented well, but soon returned under another surgeon, when nearly the same treatment was adopted, oxymuriate of mercury being substituted for the comp. calomel pill. These statements are corroborated by the books.

He is now in a good state of health, with good appetite, undisturbed rest, strong pulse, and regular bowels; he, therefore, is ordered to rub in a drachm of the strong mercurial ointment every other night—the black wash to be applied to the sore.

*25th July.* He was presented after getting rid of the mercurial taint, which had thoroughly affected the constitution for nearly eight weeks—not being allowed at any time to do more than render his gums tender.

---

V.

### EXTIRPATION OF AN INCIPIENT "FUNGOID TUMOUR."

William Stansom, æt. 61, was admitted, under Mr. Green, 23d June, 1827, with a large oval swelling above the pubes; its size is that of a large melon; it is moveable, hardish, ulcerated on its surface, and tuberculated—pressure produces pain down to the testicles, and up to the loins—any strain, as also coughing, gives uneasiness in the part. He remembers it with the earliest period of his life, and was taught to believe it to be congenital. It was no larger than a pigeon's egg, and remained so until a year and a half ago; when, from no known cause, (his health being good,) it increased with some degree of pain. Nothing was done for it until ten months ago, when its growth was very rapid, and its surface began to ulcerate—it constantly bleeds, but to no great extent; a sort of water mixed with blood, as he terms it, issues from it. His health is good, and he complains of nothing; but is willing to have it removed.

On the *29th* he was conveyed into the operating theatre, and Mr. Green, after examining the tumour, determined on leaving a granulating surface, since the integuments were in part ulcerated. He began, therefore, with a circular incision round the base of the tumour, and, in dissecting it out, found it to be perfectly anterior to the muscles, but very deep, from the obesity of the patient. The looseness of the integuments allowed of their being approximated, and the edges were retained together by two sutures—adhesive straps being applied afterwards. Nothing particular presented itself during the operation. The patient was carried to bed, and on the 5th day the wound was dressed, nothing untoward occurring in the interval. The edges of the wound had adhered for some distance on each side; but there appeared some little redness about the sutures, which were consequently withdrawn. Nothing interrupted the gradual formation of firm and healthy granulations, and he left the hospital on the 23d August, with a very small wound.

This illustrates, if any single case can, the importance of removing extraordinary growths, even though they shall not have attained a formidable character; for, here, though indolent at first, it began suddenly and rapidly to assume malignancy. True fungus hematodes is usually met with in persons, where a general disorder of the system is indicated by a peculiar unhealthy aspect—a relaxed fibre—a sallowness of the skin, which is often covered with clammy sweats—a constant troublesome cough, and general debility. But it is clear that the present case was not precisely of that character, although the fungoid and tuberculated appearances indicated a state, which could no longer exist with impunity. The robust constitution, the general good health, and especially the absence of symptoms in other parts of the body, by which a similar disease might be feared, go rather to disprove a predisposition to fungoid disease; and, if so, they go likewise to determine the nature of the present state, *viz.* a *local* disease, produced by long and continued *local* irritation. It is this tumour which requires extirpation; or, delay may occasion a degeneration of it into something very formidable. A case of glandular swelling of the neck, which occurred in the hospital, (I regret to say, too far back to allow of more than an allusion to it amongst these reports,) illustrates this; for there, delay was occasioned to the operation by endeavours to remove it by the local application of iodine. The constitution became gradually but irremediably affected, while the tumour, in a few weeks, assumed an enormous size, with all, or nearly all, the characters of the present case; and, had the health permitted even, the operation must now have been deemed out of the question—so rapidly had these changes manifested themselves.

A fear, however, seems now pretty generally excited against the harbouring of incipient disease—thus, I have seen a similar state removed from the side, before the constitution has become impaired, in Winchester Hospital, by Mr. H. Lyford; so, again, Mr. W. J. Wickham, his colleague, removed a conical crust from the lip of a girl, under the apprehension that such might prove the source of future disease. Cancers, however, are even attributed to some such irritation. A malignant disease, in general, may date its origin in the same way.

---

### VI.

### FUNGOID HIP.

THEOPHILUS WEDGE, æt. 44, was admitted under Dr. Elliotson, 31st May, with excessive pain in the left hip, shooting down to the knee—there is an increased rotundity of the buttock—walking, or any motion by which the glutæi are put upon the stretch, gives excessive pain—he has been uneasy in the part for eight months, but the hip-joint has begun to swell about six weeks only. An antiphlogistic treatment was adopted, under the supposition that some deep-seated inflammation existed, until an elastic sensation was communicated to the finger on pressure, and there was a tuberculated appearance about the crista ilii, while the rotundity of the joint was immensely increased. Mr. Green was requested to see him, the 18th July, who agreed as to the nature of the disease—he passed a lancet into one of these elastic tubercles, (convinced of its nature previously,) when a little watery fluid, tinged with blood, escaped, but not sufficient to diminish its prominence or elasticity. The case has now become more striking and of deeper interest than the former symptoms had led to; for, with the increased rotundity of the glutæi muscles, the bent position of the thigh upon the abdomen, is the easiest; and, indeed, extension now gives very great pain. This is inexplicable; but, upon reference to a case exactly similar, the post-mortem examination of which evinced a most beautiful specimen of fungous exostosis, growing from both sides of the ilium, that from the venter projected considerably into the pelvis, and, no doubt, must so have displaced its contents, as to require all the room that the relaxed muscles could afford; for here, too, the thigh was bent upon the abdomen, and at last so rigidly, that no force could displace it after death, although the joint was not diseased. The appearance of the patient, though of a robust make, is altogether unhealthy—he has a pallid face, with a yellow conjunctiva—his cheeks are thin, and his countenance anxious—he complains of weakness, want of appetite, disturbed rest, and costive bowels—the remnants of issues are upon the buttocks; in consequence of which, a poultice has been deemed the best application.

Pulv. opii, gr. j. o. n.—ol. ricini, p. r. n.

*August* 15*th*. The tubercle which was opened has thrown up some large fungous granulations, which have bled freely at different times—the sores from the issues are but little inclined to heal—he has derived rest from the opium.

---

Catherine Hayes, æt. 32, was admitted, under Mr. Green, 17th July, after having pushed her hand through a pane of glass in a drunken fit, and cut her arm about three inches above the wrist. She was in a state of syncope, as was said, from loss of blood. No pulsation was felt at the radial artery below the wound, and its division was, therefore, suspected. A tourniquet was placed upon the brachial, and she was conveyed to bed. The wound was cleaned with warm water, and as soon as re-action had come on, the tourniquet was loosened, and the artery found to be divided; the dresser then secured it above and below, and brought the edges together by adhesive straps. On the 8th day, both ligatures came away, up to which time, no untoward symptoms manifested themselves; some slight febrile attack existing for a few days only, in consequence of surcharged mammæ, which a child of eight months had been sucking at, but had discontinued to do so since her admission. Some opening medicine was given her, and her breasts were drawn, under which she got quite well. From this time, the wound gradually healed, and the hand recovered its use and sensation.

### VII.

### Dislocation of the Radius Forwards.

B. A. about 40 years of age, presented himself at the hospital, 3d June, with, as he termed it, a stiffened arm. On taking off his coat and shirt, it needed scarce a moment's inspection, even for many who had never seen a similar case, to be well satisfied of the nature of the accident—so well marked were the symptoms of the head of the radius being thrown into the cavity above the internal condyle, and resting upon the coronoid process of the ulna. The fore-arm was bent at right angles with the upper, and the hand mid-way between pronation and supination. There is a marked depression anterior to the external condyle, where the head of the radius should be, and a prominent rise upon the ulna. On endeavouring to flex the arm, the radius is distinctly felt to strike against the humerus—I say *felt*, because a dislocation of the radius and ulna backwards will give the same sensation by the condyles of the humerus striking against the bones of the fore-arm, and can be mistaken, for I have seen it so, provided the thumb be not placed upon the head of the bone during the flexion. The rotation of the radius is very limited, and the position of the hand is fixed.

Mr. Green desired his dresser to grasp firmly the upper-arm from behind, just above the condyles, while he made extension at the wrist, with slight rotation outwards, so as to supinate the hand—in about 4½ minutes the radius appeared to yield, and was felt to have regained its proper situation. Equally powerful, and more continued extension had been made by the hand, so as to depress and act alone upon the radius—but ineffectually.

The man refused to come into the hospital, and to submit to have his arm confined; but said, it was an accident he was accustomed to, and had never been confined for its cure. He left us, and never re-appeared.

A lad, by name Jesse Jessiah, was admitted, under Mr. Travers, with an injury to his left elbow, and stated, that three weeks ago he dislocated his arm; that a medical man saw him, and told him the nature of his accident; and, after making some forcible extension, charged him a guinea for its reduction—splints have been upon it ever since. Upon examination there is found to be great swelling; the fore-arm bent at a very obtuse angle; and neither rotation nor flexion can be effected to any useful extent. By observing the axis of the humerus, the condyles are evidently thrown anterior to their proper place; while from that of the fore-arm, the head of the ulna certainly, if not of the radius also, is thrown backwards. On endeavouring to flex the arm, the condyles of the humerus clearly strike against the fore-arm—there is a depression behind and above the olecranon. Messrs. Travers and Green made their examination, and agreed that there was too much obscurity from swelling precisely to determine the nature of the injury—it was clear that reduction was impossible at this state, which was hourly going on to rivet the bones to their new situations. Mr. Green thought there was a fracture of the coronoid process; so that the original appearance of displacement would return on withdrawing the extension—and thus the present state will be no extraordinary circumstance, even though a supposed reduction should have been effected. Leeches were applied and poultices, until all inflammation had subsided, and afterwards passive motion, for the purpose of making as useful a joint as the present circumstances would permit.

Two dislocations of the head of the os humeri into the axilla came into the hospital on the 2d August, within an hour of one another. They were both well marked, the head being felt in the axilla, the elbow thrown out from the side, and the deltoid muscle being flattened. Both were easily reduced in the ordinary way—one remained in the hospital for a week, but the other refused to come in at all.

---

### VIII.

### A PROTRUSION OF THE FUNDUS OF THE BLADDER WITH THE UTERUS, EXTERNAL TO THE VAGINAL ORIFICE.

CHARLOTTE HAWKINS, æt. 56, has been in the hospital several months, under Dr. Scott, but has lately complained of incontinence of urine, with very great pain in the region of the bladder. She states that, in her last confinement, she was torn, and a prolapsus uteri has inconvenienced her ever since. On examination, a laceration of the perineum extends nearly to the sphincter ani—the uterus is prolapsed, its mouth and neck being external to the labia—the fundus of the bladder seems pushed down in front, and, on endeavouring to pass the finger between them, is found connected firmly with it, so that the former never descends without the other. The repeated attempts to pare the edges of a lacerated perineum, without success, almost prohibited even its suggestion in this case. Dr. Locock thought a pessora of oak-barks bruized and enclosed in a muslin bag, would, perhaps, answer the purpose of supporting the part, and giving strength by its astringency. Very great difficulty was experienced in passing it, the vaginal membrane being much irritated, and a perfect state of misery was occasioned by its presence when introduced; for the discharge getting amongst the bark, softening it, and gaining from it a dirty colour, and an offensive smell, all contributed to excite disgust, no less than a great inconvenience to the patient. Pessories of different shapes had been tried, and all had been attended with some disadvantage. A cylindrical pessory at last was proposed by Mr. Green: it is an oblong and hollow piece of wood, with a stem attached to it, at the end of which is a ball and socket joint, to which tapes are fixed, to prevent its descent, in whatever position the body may be. This was found, after the stem was removed, to answer every purpose; not producing, as both the round or flat ones did, unequal pressure, and consequently pain and inflammation; but, being adapted to the vagina, threw the weight of the parts sustained upon a larger surface. An injection is constantly used, in order to check the discharge, no less than to correct the foul state induced by its accumulation.

SUSANNAH COOMBS, æt. 44, was admitted, under Mr. Green, 21st June, and, while in the hospital, directed our attention to a fluctuating tumour, a little larger than a walnut, on the left side of the neck. A small black spot was seen about its centre, of the size of a large pin's head—the end of a probe can be passed into it, after which a small quantity of watery fluid issues from it by pressure. There are two ulcerated spots on her shoulder, which, she informs us, were preceded by a similar tumour, which inflamed, mortified, and left the present state of parts. Her health is considerably disordered, as the sores would indicate, and the chief attention is directed to that circumstance.

My object in relating this case is, that it is an instance of an encysted tumour arising from a *sebaceous follicle,* with the circumstances which suggested the idea self-evident: for the opening on its top, which is not the product of ulceration, can communicate with the cyst in no other manner than by the neck of that follicle whose bulbous extremity is expanded to form the cyst. Some time afterwards, the tumour became inflamed, and Mr. Green, on the 12th July, said that, contrary to surgical principles, he would make an opening, for he felt convinced that a process of sloughing would take place which would be extremely painful and tedious, and the incision, at the farthest, could but hasten it. After the introduction of the lancet, such an inspissated state of the contents existed, that it was necessary to evacuate it with a probe, when the nature of the tumour could be determined by the smell alone, *viz.* the most offensive of any secretion in the body.

The part ulcerated, and she gradually got well.

---

### IX.

### A FATAL CASE OF WOUNDED LARYNX.

Thos. Wyat, æt. 36, was admitted under Mr. Green, on the evening of 14th July, with a cut throat. He appeared in a state of exhaustion, or rather of suffocation, breathing with great difficulty, and having a weak, irregular pulse, cold skin, livid lip, and glassy eye. The dresser removed the dressings from the wound, which, notwithstanding the larynx was completely divided, was entirely closed. This gave exit to a large quantity of mucus, and was followed by a speedy relief to all the above symptoms: the wound was extremely irregular. Those who brought him assured us that a very large quantity of blood was lost; yet no vessel appeared wounded. Mr. Green was sent for, and, on his arrival, desired the wound might remain open, and the dresser to stay with him the whole night, when, if unfavourable symptoms were developed, he was to be sent for again. Mucus continued to rush from the wound, obstructing the ingress of air, until 4 o'clock, when the man breathed without difficulty and slept.

On the noon of the following day, some degree of febrile action was excited, and he complained of pain in the chest, which was covered for some distance with an erysipelatous blush—he is distressed by occasional fits of coughing. An injection of jelly, strong beef-tea, and 40 drops of laudanum, was ordered.

*17th.* Nothing has interrupted the symptoms of the last report, either for the better or worse. Being incapable of utterance, he has expressed himself in writing to have been desperate in the attempt at *suicide,* but that it was his intention to submit to any restraint placed upon him for his benefit. Mr. Green fed him with the stomach syringe, and desired it to be repeated twice a day. The food was the same as the injection.

*20th.* Mr. Green ordered a third meal a day, of biscuit powder, milk, and brandy, finding a decided sinking of the pulse —indeed, he expressed himself astonished that no alteration had taken place before. The wound gapes at least an inch and a half, and rather than being inclined to heal, appears rounded off at the edges and foul, as if partly by an ulcerative, and partly by a putrefactive process—the pain and tightness of chest are gone, but he gets no rest. From this time his strength gradually sank, without any alteration of symptoms, until the 21st, when he died.

On examining the wound, the arytenoid cartilages were found to be jagged and divided; the thyroid separated both from them and the trachea; there appeared no wound in the œsophagus, as was supposed from a fit of coughing during the attempt to feed him without the syringe. No artery or vein was wounded.

---

### X.

### SPASMODIC CONTRACTION OF THE THUMB.

Sarah Fotzer, æt. 17, appeared at the surgery with a contracted thumb. She stated that, an hour previously, she had struck her thumb suddenly with a patten, which was followed by immediate pain and contraction of the thumb. It is bent inwards upon the palm of the hand; extension of it can be made, but gives pain in the part and up the fore-arm. Hirud. vj.; Lot. sp. vin.

*6th June*. She was admitted with an increase of pain up the fore-arm and the same state of the thumb. Pulv. hyd. c. scam. gr. xij.; Hirud. xij.

*8th*. Less pain, but contraction of thumb still rigid. Hirud. viij.; Cat. micæ, panis.

13*th*. Presented well.

Mr. Tyrrell has met with several cases of the same nature, when any sudden force has been partially applied. Thus, by pulling a silk handkerchief quickly through the hand, or by hitching the thumb in the key of a door while turning it suddenly, the same spasmodic contraction has occurred. His practice is to overcome the action and keep the thumb straight upon a splint, while he applies a blister to the fore-arm, which is generally successful alone.

---

### XI.

### FEMORAL HERNIÆ IN THE MALE SUBJECT.

THOS. STEERS, æt. 54, a labourer, was admitted, 12th July, under Mr. Green, with a small, circumscribed, and slightly elastic swelling in the right groin; no impulse was given to it by coughing, but it lay as if folded over the falciform process of the fascia lata, to its upper and outer side. His history is clearly, that, three weeks ago, during an attack of vomiting which ushered in a typhoid fever, he felt a sudden pain in his groin, as if something had given way; this excited his alarm for a little while, but soon was forgotten, either giving no longer pain, or vanishing altogether: yesterday, however, a renewal of the vomiting, which lasted until this morning at 12, brought the swelling into notice again, by an excessive pain. His medical attendant had tried the taxis, and bled him to syncope, but without effect. About two hours after admission he was placed in a warm bath, and the taxis again was fairly applied, his pulse not yet having perfectly rallied from the effect of the venesection. Mr. Green was now sent for, and arrived at half-past six. After obtaining the above history from the patient, and the treatment adopted by the dresser, he proceeded to examine the patient, and found that the belly was somewhat tender on pressure, and that the tumour was irreducible; and, notwithstanding the elasticity in the tumour, the compressible pulse, the absence of vomiting, or any anxiety of the countenance, he immediately proposed the operation, which was consented to.

The operation was commenced by an incision through the integuments drawn across the tumour parallel to Poupart's ligament, and by another meeting its middle at right angles. The flaps thus produced, being reflected, the cellular and fascial coverings were carefully divided, which exposed an unusual layer of fat surrounding the sac, on opening which a small quantity of serum escaped, and the intestine appeared greatly discoloured. The bistoury was then introduced upon the director, and the stricture, which was found to be Poupart's ligament, was divided, when a very large quantity more of serum escaped from the cavity of the abdomen. The intestine was returned, and the edges of the wound brought together by a suture and dressed with adhesive strap—two small bleeding vessels were secured during the early part of the operation. On his being replaced in his bed, we found him relieved, his pulse larger and more expanded, and pressure, even, gave no pain. Mr. G. left directions that, should the pain continue, fomentations were to be applied, or even leeches, according to the judgment of the dresser; but, if the pulse indicated it, venesection was to be had recourse to.

13*th*. No pain left—pulse soft and expanded—has had no relief in his bowels. ℞. Magn. sulph. ℥ij. ex m. m. omni horâ ad sedes.

14*th*. Towards the evening, his bowels were relieved—in every respect, he is doing well.

On the 15*th*, the wound was dressed, and found to be adhering in part, and the rest granulating well.

On the 17*th*, he was allowed the house diet, no symptom having appeared by which meat is counter-indicated.

From this period, he got progressively well, no alteration either in symptoms or treatment having occurred. Now, the alertness of Mr. Green will serve as an example of its importance in all cases; no less than an opportunity for specifying his reasons for adopting it. Let me first ask, what is gained by delay in these cases? Did we know the changes which are going on within the sac, so as to calculate on them with certainty, we might, perhaps, be borne out in waiting the result of other means for its reduction: for, although a spontaneous reduction has often occurred after all artificial means have failed, and, as in the present case, the inflammatory action will subside upon withdrawing the cause, yet, from the recollection that the symptoms of inflammation are sometimes extremely insidious, that the most powerful remedies have been applied without effect, and that the operation is neither a painful nor a dangerous one, we may safely conclude, that its proposition ought speedily to be made. The present case will shew how fair this view of the subject is, for effusion had taken place to a considerable amount, as the most probable effect of inflammation, although the existence of symptoms, beyond a slight pain on pressure, could not even induce its suspicion, and the protruded intestine was discoloured far beyond the expectation of Mr. Green and those around him. If irreducible, then, by the powerful remedies of bleeding and the warm-bath, carried to a proper length, Mr. Green considers a hernia but aggravated by subordinate measures; and, indeed, we cannot know when we are safe, except by such a rule.

Edward Tapner, æt. 47, was admitted 29th July, under Mr. Travers, with a painful swelling in the right groin about the size of a large walnut. It lies below Poupart's ligament, upon the fascia lata, and receives some impulse upon coughing, at its lower and inner part—it is circumscribed, and rather tense. The patient states that it appeared suddenly upon a fit of coughing, about 20 hours previous to admission, and has continued to give pain ever since. There is pain in the abdomen, aggravated by pressure, an anxious countenance, occasional vomiting, and a small, quick pulse.

On his admission, he was placed by the dresser in the warm-bath, and the taxis was applied, without effect, the tumour not yielding in the least. At 9 o'clock, Mr. Tyrrell arrived, and, in the absence of Mr. Travers, determined on operating immediately, as the only chance of relief to the patient. Consent was gained, and the operation was conducted in the ordinary way, no variety or difficulty presenting itself.

On being put to bed, Magn. sulph. ℥ij. T. opii, ♏ vj. ex Aq. menth. pip. were given, and desired to be repeated every two hours: but the bowels became very much relaxed, and the dresser gave 40 minims of laudanum; this had the effect of lessening their action. We afterwards learnt, however, that a diarrhœa had existed on him previous to the descent of the intestine.

The wound has healed without interruption, those symptoms having yielded immediately after the operation which had been urgent or indicative of inflammation. A diarrhœa has constantly been upon him, and some attention has been requisite to his general state of health, since he coughs, with a pain in his chest, and has done so for nearly two years.

The difference in the after-treatment of these two surgeons is remarkable. It is a practice with Mr. Tyrrell to stimulate the intestines by purgative medicines, immediately after the stricture is divided, although the intestine is inflamed —indeed, it is Sir A. Cooper's plan of treatment to carry this to a surprising length, and many reasons are said to support it; but Mr. Green endeavours only to soothe the part until inflammation has subsided, and, even then, is reluctant to give purgative medicine, unless the bowels have not been relieved; for if inflammation exists, he considers stimuli but calculated to increase it. Reasoning, however, will hardly decide the question when practice has proved the success of both parties. A preference can only be given after an accurate comparison of the two, which I am incapable of establishing in the present reports.

---

James Scisson, æt. 34, was admitted, 31st May, 1827, under Mr. Green, with a wound in his leg. He informs us that, 14 years ago, when a soldier in Spain, he received a musket ball in his leg—he did not perceive any pain for many minutes, but was afterwards disabled from using the limb. Hard labour has been much interrupted, and almost entirely prevented ever since. For the two first months bone was continually coming away, amounting to a dozen pieces, at least.

There is a sore at about the lower third, with irregular edges, exposing a metallic body, nearly an inch deep—there is a constant discharge, and great swelling above and below the sore, which neither pits on pressure, nor is painful. Ordered, cat. lini, and rest.

*15th June.* The swelling has subsided in great measure, and was considered by Mr. Green in a fit state for the extraction of the foreign body. Mr. G. commenced, by making a crucial incision over the wound, and reflected back the four flaps, so as to expose the whole of the body; it appeared imbedded in a cartilaginous substance which was connected with the bone. Sufficient of the ball being exposed to afford a firm purchase, Mr. Green grasped it with a stout pair of rough forceps, and, with a long, firm, and steady effort, succeeded in dislodging it. On examination, it was found to be very irregular, its rounded form being by no means preserved. The wound was lined, as it were, with cartilage, and bled pretty freely. A dossil of lint was laid upon the wound, and the patient conveyed to bed.

Bleeding continued for some short time, and the leg, above and below the wound, became extremely painful: this, however, in a few days, subsided, and a slough came away of those integuments which were divided. The wound granulated, and the limb became stronger, when gentle motion was first permitted, and afterwards the full use of the leg.

---

### XII.

### A FATAL CASE OF BRONCHITIS TREATED WITH TARTARIZED ANTIMONY.

T. CARLTON, æt. 40, a sailor, was admitted under Dr. Elliotson, 12th July, 1827. He states that, six weeks ago, he was thrown overboard, and nearly drowned, since which his present complaint has more or less troubled him. He has been constantly exposed to wet, to which he attributed the continuance of the symptoms, if not the prevention of their cure. His breathing is quick and labouring, accompanied by the mucous guggle—he complains of no pain, but a tightness and sense of suffocation—his cough is a croaking, metallic sound, attended with occasional difficult expiration—his lips are livid, skin cold, tongue brown and moist, bowels costive, pulse quick and small. Ordered, V.S. ad ℥xvj. Liq. antim. tart. ℥ss. 2dâ quâq. horâ.

*13th.* He was relieved by the bleeding, and vomited the antimony three times only. Symptoms but little altered.

*14th.* 4 P.M. Complains of intense pain round the right side—his breathing is very difficult—his feelings he described to be of suffocation, and his appearance is the same,—a cold and cadaverous skin—an almost vacant stare—aggravation of the mucous guggle—slow and feeble pulse—he complains of lightness in the head, a singing in his ears, and dimness of vision. Mr. Whitfield saw him, and ordered V.S. ad ℥xiv. and a blister to the side. He was speedily relieved, as soon as a few ounces of blood were drawn—he coughed up some frothy mucus, which rendered his breathing much easier—the surface of the body became warmer, and the pulse expanded.

11 P.M. The symptoms returned. C. c. ad ℥xiv. which relieved him. Continue the antimony.

*15th.* Improved in every respect, though the symptoms are far from being subdued. The antimony has not been rejected since the first day. V.S. ad ℥xx. Vini ant. tart. ʒvj. 2dâ q. h. Ung. ant. tart. pectori infricand.

*19th.* But little alteration. M. s. c. statim; increase the antimony to a fluid ounce.

*21st.* He is now decidedly worse. There is excessive pain, which he describes as diffused and deep-seated—the cough is deep, hollow, and leaves a grating and tearing sensation—pulse quick and small—skin dry. Hydr. submur. gr. v. 6tâ q. h. Omit the antimony.

*24th.* Symptoms much lessened—mouth sore. C. c. parti solent. ad ℥xvj. Hyd. submur. b. d. only.

*27th.* Complains again of increased pain, while there is great difficulty of breathing, and scarce power to articulate. Vini ant. tart. ʒss. M. s. ʒj. Tinct. digitalis, ♏ xx. 8is horis.

*30th.* Relieved in some measure, but, from inability to expectorate, the mucous guggle is very great—he lies upon his back, scarce observing surrounding objects, and a cold sweat is upon his skin. Emp. canth. larg. pectori.

He died on the 5th of August, without any material alteration of symptoms. Unfortunately, no effort was sufficient to prevail upon the friends to permit an examination of the body in this interesting case.

---

John Chittenden, æt. 20, was admitted, 21st July, under Dr. Elliotson, with pain, and increased rotundity of the left hip. On examination, the inflammation was clearly external to the joint. Pulse 100, and firm—skin hot and dry—thirst—bowels confined. C. c. coxæ sinist. ad ℥xx. Lot. ammon. acet. Liq. antim. tart. ʒij. 2dâ q. h.

*24th.* Has borne the antimony without even nausea, after the first dose, which he rejected. Emp. lyttæ; Liq. antim. tart. ʒiij. 2dâ q. h.

He rejected the antimony on the following day, and it was withdrawn for 24 hours. It was afterwards given again, and retained upon the stomach for several days, when matter was supposed to be formed, and the patient was seen by the surgeon. The antimony was withdrawn, and the case became of no farther interest to require its detail in these reports.

In the former case, it is clear that the large doses of antimony were borne with perfect impunity, after a while, but they had no control over the complaint. 'Tis true that the symptoms were mitigated at first, but repeated blood-letting was exercised at the same time, and I think may fairly be deemed the more efficient remedy of the two; for the benefit received immediately from one bleeding, was not at all assisted until it was repeated; and, indeed, we may as fairly conclude that, if the antimony had not been given, but some more powerful remedy, as afterwards was had recourse to, such delay, and even the life of the patient, might have been spared. Now Dr. Elliotson is endeavouring to learn, whether inflammations of the chest alone are conditions repugnant to the violent impressions of antimony, as asserted by Continental writers, who would use drachms for our grains; or whether the same state of any other part of the body is not equally opposed to its effects; or even whether antimony, in large and continued doses, is not, as many medicines are, almost inert, when the system is totally undisturbed by disease: and, as a first step to this, Dr. E. has given it in an inflammatory disease of the hip, where, likewise, it was borne, without inconvenience, for some considerable time. This idea, however, has already been suggested to the public; and M. Velpeau has gone some way to decide the question, a short account of which appeared in the Med. and Phys. Journal. He relates several cases of rheumatism, where a trial was given to this remedy in doses of from 20 to 30 grains, and his opinion of its effect, after witnessing 30 cases of its use, is, "that, in some, it produced not the least amelioration." But in a female, where bleeding and leeching had been used without benefit, 12 grains of the tartar emetic, dissolved in orange-flower water, and given in the 24 hours, such relief followed, as not to require its repetition. Now the condition produced by the antimony is not mentioned; for we must all have seen larger doses even than these given, to produce nausea—a favourable state for the cure of almost any inflammation.

Hence, we have reason to conclude, that inflammation of the chest is *not* the only condition of the body repugnant to the effects of antimony; while it remains to be proved, whether the body, in its natural state, can endure it without inconvenience, and whether its asserted deleterious effects are suppositions merely, or the result of experience and observation.

---

### XIII.

### LITHOTOMY.

Edward Rowe, æt. 24, a spare young man, with blanched skin, which he attributes to his late debauched habits, was admitted under Mr. Green, 9th August, with stone in his bladder. He had been aware of its presence two years, and Mr. G. had sounded him previous to his admission. Some opening medicine was given him; and, on the following day, the sound was introduced, and readily heard to strike against some hard body. The operation was performed in the ordinary way, and the gorget used. No bleeding took place, and some little delay was occasioned in the extraction of the calculus, which was small and smooth. The patient was conveyed to his bed, not much affected by the operation.

On the same evening, fainting, cold skin, and small pulse, came on, which lasted for many hours, and was attributable to small, but constant oozing of blood from the wound, but which, from the quantity, was not observed; the cause, however, was afterwards discovered, and he soon rallied. Nothing untoward happened after this, and he got gradually well.

The success of lithotomists is but an empty and unmeaning echo, when in computation against the method of operating, which, in my belief, is the easiest part. Health, age, and constitution, (and thus fortune, of course,) are the real difficulties to be encountered: but, amongst the opponents to the gorget, this case stands as one from the universally successful attempts of Mr. Green, who invariably uses that instrument. If danger could arise from it, some post-mortem examination should determine it, and prove beyond a doubt, what prejudice has alone suggested; but this, in careful hands, is impossible.

---

XIV.

A WOUND OF THE BRACHIAL VEIN, GIVING RISE TO SYMPTOMS AND SUSPICION OF SPURIOUS ANEURISM.

WILLIAM PAINTER, æt. 35, was admitted, 28th June, under Mr. Tyrrell, with great swelling of the left fore and upper arm. At the inner and upper third of the humerus, there is an incised wound, about half an inch long: for some distance round the wound, there is an indistinct pulsatory motion, synchronous with that of the heart—the integuments are much hardened—the fore-arm and hand are much swollen, but cold, the radial artery being scarcely to be felt—pain is excessive. Hirud xxx. Cal. gr. j. Opii, gr. ij. nocte sumend. Fot. papav.

The history of this case was, that, a month ago, his wife stabbed him, in a drunken scuffle, with a common case-knife; the part swelled immediately, but gave no pain. Little notice was taken of it until the following morning, when it became very painful, and bled profusely. Some adhesive plaster was placed upon the wound by a neighbouring surgeon, which restrained it, and fomentations gave relief: he used his arm as usual three days, but was then prevented, by excessive pain and swelling in it, which, he says, increased, occasionally bleeding, until the arm arrived at the present state, which is nearly three times the ordinary thickness. He has used purgative medicine occasionally, but had applied no leeches to the part. He has lost flesh considerably, being of robust make.

*29th.* Mr. T. was induced to call this a *spurious aneurism, following a wound of the brachial artery*, and, from the state of the limb, amputation seemed to him the only feasible operation; but even this must be deferred. Hirud. xxx. T. opii, gtt, xxx. stat. m. s.c. p. r. n. Cat. lini.

*30th.* Two very large vesicles, and several smaller ones, appear on the fore-arm and hand—sensation in the neighbourhood is very indistinct. Hirud. xx.

*2d July.* Mr. T. fancied he saw an evident thinning of the integuments round the wound, and, consequently, deemed the immediate operation the only treatment left. The consent of the patient, and approbation of his colleagues, being gained, he was conveyed into the theatre. The method of operating became then the question, for there were scarcely four inches of the deltoid left sound, while the inflammation had extended nearly close to the shoulder on either side. It was determined on to saw through the bone, just below the tubercles. Accordingly, Mr. T. began by making a semilunar incision, so as to include all the healthy deltoid in its cavity, and continued, by connecting the two extremities by another incision, carried under the arm, thus making a single flap from above; the bone was then sawed through beneath the tubercles, and the artery, which had bled very freely, notwithstanding Mr. Travers was making very firm pressure above the clavicle, was secured with some little difficulty. A suture was passed through the integuments, and the patient conveyed to bed. After a short time, finding that no hemorrhage had taken place, Mr. Tyrrell dressed the wound. From the loss of blood, the poor fellow now had a cold skin and small pulse, from which state he did not rally until the 2d day. Opium and wine were ordered.

*4th.* Skin hot, and diarrhœa. Mist. effervesc, c. T. opii, gtt. v. 4tis horis. Porter, ℔ss.

*5th.* Very little alteration. Porter, ℔j. Eggs, ij. Cal. gr. j. Op. gr. ss. n. s. Mist. efferv.

6, P. M. Omit the porter. Vini rub. ℥vj.; Mist. cretæ post sing. sed. liq.; T. opii, gutt. xx. statim.

*9th.* Wound was dressed; and, but for some small sloughs, was looking pretty healthy—he was not materially altered in other respects.

*10th.* Last evening his stump became painful—he complained of nausea and occasional retching—he is to-day in a very low state—his countenance is cadaverous, and pulse rapid and feeble—he answers with reluctance, and notices nothing. The parts surrounding the wound are swelled, and the dressings are in part removed—brandy was ordered hourly. No change but that of evident sinking took place after this report. He died this evening.

The amputated limb exhibited, on examination, not an aneurism from wound of the artery, but a wound simply of the accompanying vein. With respect to the other part of the limb nothing particular was found—thickening of the integuments round the wound for some distance—œdema of the lower arm, and superficial gangrene.

---

### XV.

### ICTHYOSIS.

Wm. Goodwin, æt. 11, applied at the hospital as an out-patient, under Dr. Roots, 12th July, 1827. The whole of his limbs, excepting the joints, were covered with a calcareous scale, which was hard, of a darkish grey colour, and intersected into small square meshes. He has always enjoyed good health, though of a well-marked scrophulous diathesis, but has never been free from the present complaint since he was a year old.

His bowels are confined, so that a week will sometimes pass without his procuring a stool; he has thirst at present, and a bad appetite; his urine is in full quantity, light in colour, and without sediment—his body is constantly itching. Ordered picis liquid, ʒss.; Pulv. glycirrhizæ, q. s. ad massam formand. quam in pil. xl. divide cap. iij. 6tis horis; Balneum tepid altern. auroris.

This remedy was continued for about five weeks, when he complained of sickness to a great degree, so that it could no longer be borne on the stomach for any length of time, and the impure prussic acid was prescribed. The effects of this equally nauseating medicine I have not been able to collect, nor do I know the object of its application. 'Tis true, that the scales are now pretty generally cleared from the skin, but it must be supposed that the bath was more influential in removing them than the pitch; and, of course, as far as the constitutional disposition is concerned, little amendment can yet be visible. Dr. Mason Good alludes to this treatment, but says, that he never heard of any permanent good effect from it; for, as soon as *local* remedies even are withdrawn, the scales have re-appeared.

The pathology of this disease seems to be a metastasis of the secretion of the calcareous earths. In like manner, Dr. Good relates two cases, where calcareous matter was secreted with the saliva and urine, and where, upon examination of their bones after death, little more than cartilage was found, of which "the scalpel, with very little force, ran through the hardest." But a simple vitiated secretion is seen in mollities and fragilitas ossium, of which the present case can be but a variety.

---

### XVI.

### DIFFUSE INFLAMMATION OF THE CELLULAR MEMBRANE AFTER BLEEDING.

Sarah Groombridge, a plethoric girl, of 21 years, was admitted, 23d of July, under Mr. Tyrrell. She complains of pain and heat extending above and below the elbow-joint to a considerable distance—there is much swelling, so that the arm is nearly twice its natural size, affording a pit on pressure, and producing some pain by the motion of the fingers. Two days after being bled, she states that her arm began to be painful, and to swell. Poultices and opening medicine were employed, and it gradually increased to the present state, being four days from its commencement. There is some constitutional excitement, the skin is dry, pulse full, and tongue white; there is thirst, and her bowels are constipated, unless moved by medicine. Ordered, Hirud. xxx.; Lot. plumbi subacet. dilut. c. cataplasm miscæ, panis. Hydr. submur gr. v. hac nocte sumend.; M. s. c. cras mane sumend. et rep. ad sedes liquid.

*24th.* Excitement increased, and local symptoms more severe. Hirud. xx. cat. lini.

*25th.* Improved—she has slept, and her skin is moist. Hirud. xxx.

*27th.* Pain and redness of the arm are gone, but have left considerable swelling, which pits on pressure. Some watery fluid, resembling pus in colour, has escaped from the puncture of the venesection. All fever is gone.

*30th.* A second puncture was made, and a large quantity of thin watery discharge was evacuated. Some hardness left.

*2d August.* Emp. saponis to be applied up the fore-arm.

This case, although it may not have been so severe as is often presented to us, will serve to illustrate the usual practice at this hospital of treating similar cases. Mr. Tyrrell has a great objection to the decided measures proposed by Messrs. Earle and Hutchison, *viz.* of incisions; for, in his opinion, the only real benefit is the loss of blood; but, then, we have no command over the quantity, and patients have repeatedly been known to sink from that cause; while, by leeches, the same object is arrived at, with comparative certainty, and its success has been fully established. With Mr. T. this plan has never failed, where he has had the commencement of the attack to deal with.

---

### XVII.

### A FATAL CASE OF COMPOUND FRACTURE OF THE TIBIA AND FIBULA.

William Brown, æt. 70, a labourer, was brought into the hospital, 27th June, 1827. On examination of the left leg, was found a fracture of the tibia and fibula, with obliquity downwards and outwards, a wound of about ¾ of an inch was produced by the protrusion of the lower portion of the tibia, and a small piece of the upper portion lay detached within the wound; there was no difficulty in reducing it, and but little blood issued from the wound; a small dosil of lint was laid upon the orifice, and the leg was placed between two splints, laid upon the back, and raised at the heel. In the evening some swelling was perceived, and the patient complained of great uneasiness—the splints were loosened, and a spirit lotion to the whole leg; after which, nothing untoward occurred until the evening of 1st July, when the parts surrounding the wound appeared much inflamed—pulse raised, skin hot, and thirst—his bowels were confined. Ordered s. c. cochl. iij. 2dis horis donec alvus respond.

*2d.* A small sloughy spot covers the wound, and an inflammatory blush extends over the whole leg—pulse is full, but very compressible—tongue brown, skin hot—18 leeches were ordered; a poultice over the wound; the spirit lotion to be continued; and the splints to be entirely removed. Rep. m. s. c.

*3d.* Sloughing has continued. P.

*4th.* The lower portion of the tibia is exposed—slough increased. The leg was to-day placed in Sir A. Cooper's fracture box, which operation produced no pain.

*5th.* Some healthy discharge—fever gone—pulse much lessened in volume, and quicker. Porter ℔j. quotidie.

*7th.* Limb looking better—inflammation subsiding, but his bowels are very relaxed, and he is scarcely able to retain his stools. Mist. cretæ c. to be taken after every liquid stool. Port wine, ℥ij. and porter.

*9th.* Debility increased—bowels still purged—tongue brown—pulse quick, and very compressible. Omit the porter, in the fear that it gave rise to the diarrhœa. Wine, ℥iv. ℞. T. opii gtts. x.; Ammon. carb. gr. v.; Mist. camph. ℥iss. 6tis horis s. sago and syrup.—Continue the poultice.

*12th.* Restless—pulse irregular—vital powers evidently sinking. Brandy ad libitum.

From this report he gradually sunk. His age and constitution were both points of great consideration. The same degree of inflammation in a young and robust subject might have been combated with blood-letting, but certainly could not have been treated without its employment.

The pulse here was of that full, but powerless volume, which indicated a flabbiness rather than excess of action in the heart and arteries, and, as such, the small abstraction of a few ounces of blood would have been attended, perhaps, with a fatal syncope. The case might be contrasted with a similar injury in a middle-aged man, where a great destruction of parts was attended with very little suffering of the constitution.

John Ellis, æt. 22, was admitted, after having received a blow upon the upper-arm from the handle of a winch, while nearly a ton weight was hanging to it. On examination, a fracture was found, extending obliquely downwards and outwards, through the shaft of the humerus, at its lower third—a small wound communicates with the lower portion—there is not much tumefaction. The wound was dressed with adhesive plaster, and the limb supported by two splints, one on the inner and the other on the outer side. A cold lotion was ordered, and the fore-arm kept at right angle with the upper, raised on a pillow.

A few days after, he complained of uneasiness, for which the splints were loosened; but, the wound being on the under side, was not examined, from time to time, so regularly as would have been permitted, had it been situated elsewhere. On the 7th day, great pain was experienced, and febrile symptoms developed themselves; this was considered to arise from the riding of bones, since the splints could give no support, from their looseness. The patient was, therefore, directed to sit up, when the wound, which was in a foul condition, was discovered to be surrounded with a large slough; it was, therefore, dressed with the red precipitate ointment, and supported by four well padded splints, lightly applied. He was desired to remain in the semi-erect position, and suspend the elbow, while he supported the fore-arm only, at right angles with the upper, in a sling. This gave him great ease; and, from this time, the splints were gradually drawn tighter, as the inflammation subsided, and the sore would bear pressure. His health suffered but little, and all excitement subsided so soon as the irritation was withdrawn.

It must be desirable, in compound fractures, that rest and the recumbent posture should be enjoined until all inflammatory symptoms shall have subsided, even in the upper extremity, where motion need not be used: and the present case is an exception, only because the wound being on the under side, the limb was necessarily disturbed at each dressing; and, from the riding of the ends of the bone, in consequence of its obliquity, the inflammation must thereby be kept up, if not altogether excited. But the extension which the weight of the elbow afforded, got rid of the real source of irritation, and allowed of sufficient pressure to support the limb, and effect a comfortable apposition of the broken ends. Nothing afterwards retarded his gradual recovery.

---

### XVIII.

### A CASE OF POISONING BY ARSENIC.

A woman, apparently about 60 years of age, was brought into the hospital, under Dr. Elliotson, in a state of exhaustion; her pulse was scarcely perceptible, skin cold, and pupils fixed—she was incapable of answering when spoken to—pressure in the epigastric region gave excessive pain, under which she would writhe and exclaim some imperfect sound—her breathing was not at all effected—her tongue was dry, and she appeared to wish for drink constantly.

Those who brought her knew little or nothing about her, but informed us that, on entering a neighbour's kitchen, about an hour ago, she had begun to eat some bread-crumbs, which were lying in a plate mixed with arsenic, for the purpose of destroying black-beatles, but would not desist on being warned of it.

The stomach-pump had been used, sufficiently merely to obtain a specimen of the contents of the stomach, which, together with the remaining crumbs of bread, were analyzed by Dr. Bruton, and found to contain arsenic. Another pump was procured from the hospital, the former being out of repair, and a second attempt was made by the dresser when the stomach was emptied and filled several times with luke-warm water, until the fluid came unchanged by any admixture from the stomach; it having received a tinge of blood each time. Nearly two hours had now elapsed since the accident. The treatment became then a matter of consideration. Inflammation existed, as was clear from the excessive pain about the region of the stomach, no less than from the blood which stained the water. The state of exhaustion, however, forbade blood-letting; but a large dose of opium was determined on, and mucilaginous fluids; a glyster of ol. ricini and ol. olivæ was given; leeches also were ordered, but she sank before they could be applied.

---

### XIX.

#### A CASE OF SUPPOSED FUNGUS OF THE BRAIN BENEFITED BY IODINE.

WILLIAM HICKS, æt. 44, admitted, under Dr. Scott, 12th July, 1827, complains of imperfect vision of the left, and total darkness of the right eye. The recti muscles are likewise partially paralysed, their motion being very limited, if not altogether gone: thus he is able only to turn the right eye upwards, and the left a little to the side in addition; no adaptation, therefore, of the eyes can take place, and he sees objects doubled beyond a certain focus, the axis of the one crossing that of the other at a certain point. The pupils are small, and do not contract to the stimulus of light. He has also a numbness of the lower extremities, with occasional flashes of pain, extending from the foot to the thigh—there is such debility of these limbs, that he drags them after him in walking, and often misses his step. His hands, likewise, partake of this debility, for they are not capable of offering any degree of force.

He has passed a sedentary life, being a weaver, and has been constantly the subject of head-ache for these two years past—bleeding, however, generally relieved it.

Dr. Elliotson, in the absence of Dr. Scott, said, he considered these symptoms to arise from the presence of fungoid growths in the cranium; he, therefore, ordered T. iodinæ, ♏. x. ter die; Abrad. capilli et app. ung. iodinæ capiti bis die.

The patient continued these medicines, gradually increasing the former to xxv. ♏. until the 5th of August, when the stomach became irritable, and it was withdrawn. Its use had been attended with decided benefit—the eyes are less dark, and he can move them in almost any direction—his feet are stronger, less numbed, and but seldom affected with those stinging pains complained of on his admission. Blisters were made use of afterwards, but almost immediately upon the abstraction of the iodine his pains returned, his walking was attended with the same uncertainty as at first, his sight became dimmed, and he complained of head-ache. The subsequent treatment I was unable to learn.

G. WICKHAM,
*Winchester.*

---

### HOSPITAL REPORTS.

REASONS, which are sufficiently set forth in the Address at the close of this Number, induce us to state that, in future, this Journal will be entirely dedicated to the subject of REVIEWS of BOOKS and JOURNALS. Our HOSPITAL-REPORT PRIZE has called forth a number of competitors beyond our most sanguine expectations; and we have, at this moment, reports enough in our hands to fill a large volume. To several of the competitors we shall adjudge the Prize, (as will be seen in the list) although we are unable to give insertion to the Reports themselves. We claim the privilege, however, of handing them over for publication to some of our cotemporaries. To these cotemporaries, we have recommended the example of offering Prizes for Hospital Reports; and we have reason to believe that they will adopt it. We return our sincere thanks to the Candidates who have favoured us with their labours; and we earnestly entreat all who have opportunities of observing the events of HOSPITAL PRACTICE, to favour us with the correction of *erroneous* Hospital Reports, wherever they may be published, in order that truth may predominate over error. The HALF-MONTHLY Fasciculi, in which form this Journal will henceforth be published, will afford an immediate antidote to the poison of *false reports;* and if surgeons do not embrace this medium of correcting falsehoods, they deserve all the evil consequences that may attach to the propagation of error. The statements must be *authenticated to us:*—but the contradictions must invariably appear in our own language. No original papers, of any description, can come within the three sheets of which each Fasciculus of the Journal will be composed. EXTRA-LIMITES must be at the expense of the individual claiming that medium.

